# New Tests to Measure Individual Differences in Matching and Labelling Facial Expressions of Emotion, and Their Association with Ability to Recognise Vocal Emotions and Facial Identity

**DOI:** 10.1371/journal.pone.0068126

**Published:** 2013-06-28

**Authors:** Romina Palermo, Kirsty B. O’Connor, Joshua M. Davis, Jessica Irons, Elinor McKone

**Affiliations:** 1 School of Psychology, and ARC Centre of Excellence in Cognition and its Disorders, University of Western Australia, Crawley, Australia; 2 Research School of Psychology, The Australian National University, Canberra, Australia; 3 ARC Centre of Excellence in Cognition and its Disorders, The Australian National University, Canberra, Australia; University College London, United Kingdom

## Abstract

Although good tests are available for diagnosing clinical impairments in face expression processing, there is a lack of strong tests for assessing “individual differences” – that is, differences in ability between individuals within the typical, nonclinical, range. Here, we develop two new tests, one for expression perception (an odd-man-out *matching* task in which participants select which one of three faces displays a different expression) and one additionally requiring explicit identification of the emotion (a *labelling* task in which participants select one of six verbal labels). We demonstrate validity (careful check of individual items, large inversion effects, independence from nonverbal IQ, convergent validity with a previous labelling task), reliability (Cronbach’s alphas of.77 and.76 respectively), and wide individual differences across the typical population. We then demonstrate the usefulness of the tests by addressing theoretical questions regarding the structure of face processing, specifically the extent to which the following processes are common or distinct: (a) perceptual *matching* and explicit *labelling* of expression (modest correlation between matching and labelling supported partial independence); (b) judgement of expressions from *faces* and *voices* (results argued labelling tasks tap into a multi-modal system, while matching tasks tap distinct perceptual processes); and (c) *expression* and *identity* processing (results argued for a common first step of perceptual processing for expression and identity).

## Introduction

Faces display a range of social information, and are used to infer the identity, age, gender, gaze direction, and emotional state of others. Here, we are interested in assessing potential variation in abilities in the typically developing, non-clinically selected, population (i.e., “individual differences”).

In regards to facial *identity*, it has recently been established that, perhaps surprisingly, such normal-range individual differences exist. That is, it is not that case that we are all “experts” at recognising faces. Tests have been developed with high validity and reliability that measure and reveal these large, reliable and stable individual differences in face identity ability across the typical population (e.g., [1]–[5]). Moreover, it has been demonstrated that such tests can then provide useful theoretical insight. For example, individual differences correlations have been used to demonstrate that facial identity recognition ability is largely heritable, as a specific ability independent of general intelligence [6]–[8], and that an individual’s face identification ability is related to their psychosocial functioning (e.g., poorer facial identity recognition is associated with increased social anxiety [9]).

In addition to identity, faces also convey facial *expressions of emotion*. The ability to accurately and efficiently recognise facial expressions is fundamental for many typical interpersonal interactions, facilitates social cognition [10], and may promote successful social interactions, such as increased pro-social behaviour [11]. It is well established that there can be clinical-level deficits in the ability to process facial emotions in certain disorders, but the situation regarding individual differences in the typical population is less clear, and currently available tasks are not necessarily ideal for measuring within this range. The properties of previously available tests are summarised in Table 1.

**Table 1:** Summary of some previous tests of facial emotion matching and labelling.

Test (authors)	Emotions included in test	Reliability^a^		Mean % Accuracy (SD)	No. of trials		Stimulus presentation time		Number of individuals displaying expressions
***Matching***									
Facial affect discrimination [12]	Six basic emotions (anger, sadness, fear, disgust, happiness, surprise) and neutral	Not reported		88.71 (7.67)	42		500 ms		3
Face discrimination [13]. Judge whethertwo posers displayed the same ordifferent emotions.	Anger, sadness, fear, disgust, happiness, pleasant surprise, unpleasant surprise, interest	Range for a set of discrimination tasks was.39 to.63		82.14 (7.86)	28		5 seconds		28 (14 female)
Expression matching [14]. Choose whichof four faces displayed the sameexpression as the target face.	Six basic emotions	Not reported		95 (1.5)	24		Until response		24 (12 female)
Emotional odd-man out [3]. Judgewhether the expression of a centrallypresented face differed from the left andright distractors.	Six basic emotions	0.46 (0.76 RT)		88 (7)	30		Until response		30 (15 female) targets; 60 distractors (30 female)
***Labelling***									
Facial affect identification [12]	Six basic emotions and neutral	Not reported	84.33 (6.07)			21	500 ms	3	
Face identification [13]	anger, sadness, fear, disgust, happiness, pleasant surprise, unpleasant surprise, interest	range for a set of identificationtasks was.58 to.70.	73.13 (11.25)			32	20 seconds	32 (16 female)	
Diagnostic Analysis of Nonverbal Accuracy Scale (DANVA) [15]	Happy, sad, angry, fearful, neutral	0.88 (0.84 test-retest)	85.31–91.25 (5.31–7.81)in children aged between6–10 years			40 (8 neutral not scored)	1000 ms	8 (4 females, half were children)	
Japanese and Caucasian Brief Affect Recognition Test (JACBART) [16]	Six basic emotions and contempt	0.82–0.89 (across 4 tests)	68–76 (13–18)			56	200 ms	Not reported	
Emotion Hexagon [17]	Six basic emotions (morphed blends)	0.92 (split-half reliability)	89.98 (7.93)			150 (30 stimuli and 6 stimulinot scored)	5000 ms	1 (male)	
Ekman 60 Faces test [17]	Six basic emotions	0.62 (split-half reliability)	84.40 (8.40)			60	5000 ms	10 (6 female)	
Context-free Expression Labelling [14]	Six basic emotions and neutral	Not reported	0 (2)			28	Until response	28 (14 female)	
Facially Expressed Emotion Labelling [3]	Six basic emotions	0.59 (0.70 RT)	78 (9)			30	200 ms	30	
Facial Expression Recognition Task [18]	Animated morphed blendsof the six basic emotions(except surprise)	Not reported	63.50 (17.48) in children aged 10 years			160	Not reported	2 (1 female)	
Multimodal Emotion Recognition Test(MERT) [19]	Hot anger, cold anger, panic fear, anxiety, despair, sadness, elation, happiness, contempt, disgust	0.56 (test-retest for static poses); 0.78 (test-retest for entire test).6-week interval.	61 (7)			120 (30 audio/video, 30 video only, 30 audio only, 30 static poses)	2 seconds face display	12 (6 female)	

a Cronbach’s alpha unless test-retest or split-half reliability noted; Accuracy unless RT noted.

### A Need for New Tasks

**Figure 1:**
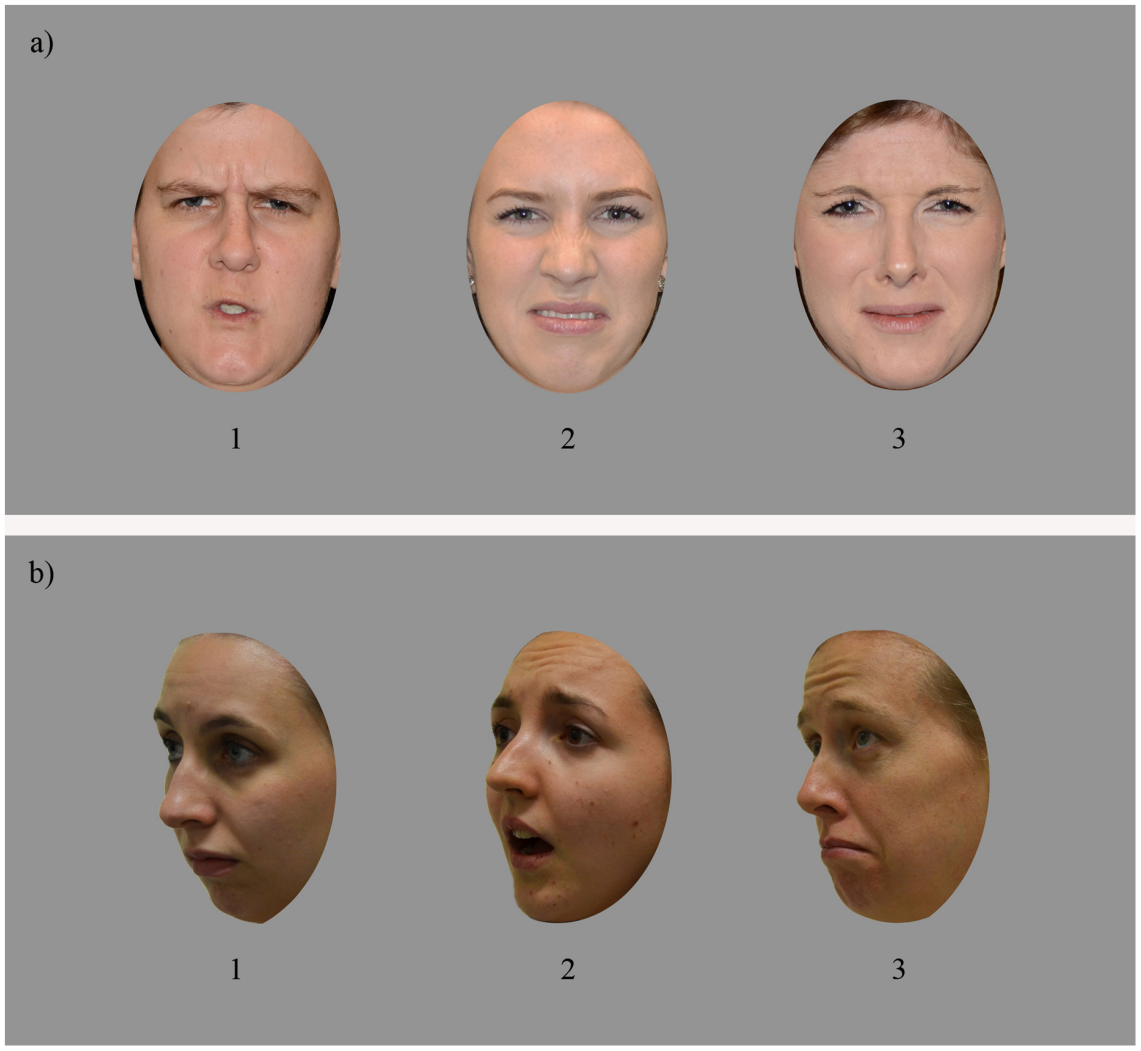
Example trials. **a)** Facial emotion-matching task with front-view images of expressions of anger (#1) and disgust (#’s 2 & 3), **b)** Facial emotion-matching task with left-facing three-quarter-view images of expressions of fear (#2) and sadness (#’s 1 & 3). Note that these face images are examples and were not used in the actual tasks.

In the present study, our first aim was to develop two new tests of facial emotion processing. The *emotion-labelling* task developed for this study, like many previous tests, gave participants a set of emotion terms (anger, happiness, disgust, sadness, fear, and surprise) and instructed them to choose the label that best reflects each facial expression displayed. In the *emotion-matching* task (inspired by the “emotional odd-man-out” task developed by Herzmann et al. [3]), each target face used in the labelling task was placed in a triad with two other faces displaying another expression with which it is commonly confused (e.g., an angry target face was paired with two disgusted distractor faces, see Figure 1) and participants were simply asked to judge which face displayed the discrepant emotion. The rationale behind our test development was as follows.

First, we wanted tests of both *matching* (specifically, an odd-expression-out task) and of explicit *labelling* of the facial emotion. Theoretically, these tap potentially different processes, in that matching requires expression *perception* but does not require verbal labelling of the facial emotions, while labelling requires initial expression perception (as with matching) but additionally requires later processes involved with assigning a verbal label to the emotion.

Second, we wanted the matching and labelling tests to use equivalent face items (specifically, all faces from one database, in this case the Karolinska Directed Emotional Faces database [KDEF [20]]; plus the target faces for the labelling task were the targets of the 3AFC odd-man-out task). This has the advantage that differences in experimental results between the tasks can then be most clearly assigned to an origin in different theoretical components tapped by the two tasks (purely perceptual, versus perceptual-plus-labelling) rather than to face-appearance differences. Many of the labelling tests in Table 1 do not have equivalent-item matching tasks available.

Third, we wanted tasks that have good internal reliability, while including all six so-called ‘basic’ facial expressions (anger, disgust, happiness, fear, sadness, surprise [21]) in the test. Logically, this is possible only if all six expressions tap common mechanisms. In older literature, it was common to suggest that certain expressions tapped mechanisms specific only to that expression. For instance, it was often argued that the amygdala had a unique role in processing fear, and that certain clinical disorders produced specific deficits in fear processing (e.g. [22]). More recently, however, it has been shown that the amygdala responds to all facial emotions (see review by Adolphs [23]) and claims of fear-specificity are not always supported in clinical disorders (e.g., for psychopathy see [24]). This leads us to expect that achieving good internal reliability, while including all six basic expressions, is feasible. Internal consistency (Cronbach’s alpha) values of above.6 [3] or.7 [25], [26] are generally considered satisfactory. As can be seen in Table 1, not all current tests report reliability estimates (e.g. [15], [18]) or, where they are provided (and accuracy is the dependant variable), reliability estimates are not as high as would be desirable (e.g., 0.62 in Ekman 60 Faces [17]; 0.46 in Herzmann et al’s [3] emotional odd-man-out task and 0.59 in their facial expression labelling task).

Fourth, we aimed to set task difficulty such that scores were suitable for examining individual differences in the normal range. Some of the tests in Table 1 show high reliability but were developed with the aim of contrasting the performance of individuals with disorders – either neurodevelopmental (e.g., autism, William’s syndrome) or acquired (e.g., traumatic brain injury) – with that of individuals from the typical population. The primary aim of these tests is thus to diagnose performance categorically, as being of either a normal or a pathological level, with no consideration given to individual differences within the typical population. As such, the performance of typical individuals is often at, or close to, ceiling, which limits the range to observe normal-range individual differences in ability (e.g., tests by Croker & McDonald [14]; Emotion Hexagon [17]). In contrast, we aimed to set our task difficulty such that mean performance of typical individuals was well below ceiling.

Fifth, we wished to ensure validity. Test validity is defined as the degree to which the test is measuring what it is intended to measure [27]. In this case, we wanted to ensure that it is naturalistic facial expression processing that is driving performance rather than any other aspect of the face stimuli. The Japanese and Caucasian Brief Affect Recognition Test (JACBART [16]) displays acceptable reliability (Table 1) but it is mainly a measure of the recognition of “micro-expressions”, which are of extremely short duration compared to typical facial expressions [28]. Other tests, including the Diagnostic Analysis of Nonverbal Accuracy scale (DANVA [15], [29]) include only a few basic expressions (rather than all six), displayed by only a limited set of individuals. Computer morphing software has also been used in several tests to create blends of confusable facial expressions (e.g., mixing angry and disgusted expressions) to make it more difficult to recognise the expression (e.g., the Emotion Hexagon [17]). However, the expressions presented in such stimuli may be impossible to produce or observe in natural faces and thus lack ecological validity.

In the present study, we adopted the following approaches to maximise validity. First, we used photographs (not morphs), and these were of a large number of individuals, displaying all six basic facial expressions (anger, happiness, disgust, sadness, fear, and surprise [21]), presented in both full-face and three-quarter poses (Figure 1). Second, we screened the results of individual items to remove any for which there was evidence that the target expression was consistently misperceived by observers. This is important because, in all these tests, expressions are posed by actors rather than recorded in natural environments (to ensure matching of photograph format), and acting quality may vary (e.g., a supposed “fear” expression may, in fact, look more like surprise). Third, we assessed divergent validity, by determining the correlation of our new tests with nonverbal IQ, with a lack of correlation indicating that individual’s test performance does not reflect merely general cognitive skill. Fourth, we assessed convergent validity, to the extent this was possible, by correlating our new labelling task with a previous widely-used labelling test (the Emotion Hexagon). Note that convergent validity is intrinsically difficult to fully assess: theoretically we do not necessarily expect strong correlations between our two new tests (because one requires perception and the other perception-plus-labelling); and even for labelling, we would expect correlations with previous labelling tasks to be falsely reduced by low reliability in some tests (Table 1), and/or lack of range where tasks are more suitable for studies with clinical samples rather than individual differences (including the Emotion Hexagon). Finally, we examined whether our tasks show clear inversion effects (i.e., superior performance on the tests when the faces are upright as compared to inverted). Perception of expression in whole faces is strongly affected by inversion (e.g., as evidenced by the Thatcher illusion [30]). Thus, strong inversion effects would suggest that participants were relying on natural facial expression processing mechanisms rather than simply using low-level cues to perform the tasks (e.g., a label of “happy” applied simply because teeth were displayed). This approach of using the presence of inversion effects to establish validity has previously been used in development of tests of facial *identity* recognition (i.e., the Cambridge Face Memory Test [2]).

### Theoretical Questions Addressed with our New Tests

Having developed new tests, our second aim was to demonstrate that the tasks could be used to provide insight into theoretical issues about the cognitive structure of face processing. We address three specific questions, in each case using the logic that when correlations are examined within the non-clinical typical population, high correlations between tasks indicate that the two constructs under consideration access common processing mechanisms, while low correlations indicate that the constructs access independent processing mechanisms. Previous literature has left our three specific questions open, as follows.

#### 1. Relationship between facial emotion perception and facial emotion labelling

As we have noted, matching tasks might be more purely perceptual than labelling tasks, as matching allows participants to discriminate between expressions on the basis of visual properties alone [31]. Labelling tasks require verbal categorisation, and so also place additional reliance on the individual’s emotional vocabulary, whereas matching tasks require no explicit verbalisation of expressions. Some have argued that language can be used to assist emotion recognition, by providing a context which limits the number of potential emotional states that stimuli could be displaying (e.g. [32]; but see [33]). It has also been suggested that a greater cognitive load is placed on working memory when performing labelling tasks than when performing matching tasks [34]. Thus, a question of theoretical interest is the extent to which face emotion perception and face emotion labelling rely on common or distinct processes.

We found only two previous studies which have correlated the ability of normal-range, non-clinical participants to match and label expressions. Addington and Addington [12] used a labelling task in which participants selected which label (e.g., happy, sad) best matched each basic facial expression, and a matching task in which two expressions were shown and participants judged whether the emotions displayed on both faces were the same or different (see Table 1 for more details). A significant correlation was shown between the two tasks (*r* = .48, *N* = 40), arguing for some degree of shared processing. However, as reliability, and thus the maximum possible correlation, is unknown for these tasks, the theoretical question cannot be fully addressed by this study (i.e., it is not clear whether the correlation reflects partially or completely shared processing). Croker and McDonald [14] used a similar labelling task, but a different matching task in which participants selected the correct match for the target emotion from four other facial expressions. They did not find a significant correlation, potentially suggesting independent mechanisms. However, performance was close to ceiling (Table 1), and the sample size was also small (*n* = 15). In the present study, we re-assess the matching-labelling relationship, using our new tests, with a relatively large sample size (*N* = 80), and calculating reliability and thus upper bound correlations. Comparison to upper bound is important because the maximum possible correlation is often well below 1.

#### 2. Relationship between processing of facial and vocal emotions

In addition to facial expressions, emotions can also be conveyed via vocalisations and body postures, and in everyday life facial and non-facial cues are often combined (e.g. [35], [36]). An important question is whether the ability to recognise emotion from one such cue (e.g., faces) is correlated with the ability to recognise emotion from another cue (e.g., voices). That is, whether people who are poor (or good) at recognising emotions from faces are likely to also be poor (or good) at recognising emotions from other modalities. Such an association would suggest a common multimodal stage of processing, in which emotions are processed similarly regardless of their mode (visual, vocal) of presentation.

In clinical cases, some studies have found that patients who have difficulty recognising emotion from faces also have difficulty recognising emotion from voices (e.g., fronto-temporal dementia [37]; autism [38], [39], suggesting a common system for processing emotion [39]. However, in contrast, Adolphs and Tranel [40] found that patients with amygdala lesions were impaired at recognising facial emotions but not impaired with vocally expressed emotions, suggesting distinct systems.

Turning to studies in the typical population using an individual differences approach, Borod and colleagues [13] assessed emotional prosody with neutral sentences spoken in various emotional tones. They found a significant positive correlation between emotional prosody matching (judging whether two subsequently presented sentences conveyed the same emotion) and emotional face matching (judging whether two faces displayed the same emotion) (*r = *.35), and also between emotional face labelling and emotional prosody labelling (*r* = .58, *N* = 100). However, a limitation of this study was that faces were not matched to the ethnicity of the participants (who included individuals of European, African-American, Hispanic or Asian descent) and evidence suggests that individuals are better at judging the facial and vocal emotions of individuals of the ethnic or cultural group to which they belong (in-group) than out-groups (e.g [41]). In addition, the vocal stimuli were English sentences and 39% of the participants were not native English speakers. Out-group participants and/or non-native English speakers may have been more likely to perform more poorly across both tasks, which may have resulted in two separate sub-samples of participants; this can lead to distinct clustering of data points, which can inflate correlations [42].

Scherer [43] also confirmed a significant, albeit smaller, correlation between facial and vocal emotion labelling (*r* = .24, *N* = 1,264). Bänziger et al [19] also present relevant data from the newly developed Multimodal Emotion Recognition Test (MERT), which involves the labelling of: static facial expressions, dynamic facial expressions, emotional vocalisations, and dynamic facial expressions with an accompanying emotional vocalisation (see Table 1 for more details). There were modest significant correlations between the ability to label emotional vocalisations and the ability to label static and dynamic facial expressions (*r* = .41, *r* = .28 respectively, *N* = 72). However, factor analysis suggested that visual (still and dynamic face labelling) and auditory (labelling emotional vocalisations with and without facial expressions) emotional processing should be seen as separate factors.

Here, we examined the relationship between our face tasks and voice emotion labelling in typical adults via a task developed by Calder, Keane, Lawrence, and Manes [44], in which participants heard five digits, between 1 and 9, read with different emotional tones. A similar task has previously been used with adults with autism, and their performance on this task was significantly associated with their performance on facial emotion labelling (*r* = .65, *n* = 23) [38]. Given this, and the studies of Borod et al. [13] and Scherer [43], we expected to observe a relationship between the *labelling* of vocal emotions and the *labelling* of facial emotions in typical adults. For the first time, we were also able to compare this to the upper bound. Further, an open question concerned the nature of the association between *labelling* of vocal emotions and *matching* of facial expressions. To the best of our knowledge this has not previously been examined. A correlation between these two tasks would suggest early common processing of emotional content.

#### 3. Relationship between processing of facial emotion and facial identity

The third question we investigated was the extent to which people’s ability to recognise *expressions* from faces was associated with their ability to recognise *identity* from faces. Popular cognitive [45] and anatomical models [46] suggest that the processing of identity and expression diverge at an early stage of face perception, which would predict a very weak or no correlation between face emotion and face identity tasks. However, a careful review and analysis of more recent computational modelling and neuroimaging data by Calder and Young [47] (also see [48]) argues that a common visual route is used for some aspects of facial expression and facial identity recognition, which would predict a significant correlation in abilities.

Previous individual differences studies examining the strength of this correlation have used the Benton Facial Recognition Test matching task [49] to measure identification ability. Two studies have reported a significant correlation between the Benton and facial emotion-labelling (*r* = .44; *r* = .35) but not facial emotion-matching (*r* = .08; *r* = .14) ([13] [12], respectively). If both facial identity and facial expression are initially processed via a common route (i.e. [47]), we would have expected an association between the emotion-*matching* tasks and the Benton, because matching more purely taps initial perception than the labelling task. In fact, it is theoretically challenging to explain why there would be an association between the Benton and the emotion-labelling task (which would presumably tap later emotion processing) but not between the Benton and the emotion-matching task. One possible reason for the lack of association may be that the emotion-labelling tasks were more sensitive to individual differences than the emotion-matching tasks (see Table 1 for evidence that this may be the case for [13]). Thus, here we re-examine this question with our more sensitive tasks.

An additional limitation of the previous studies is that performance on the Benton can fail to adequately measure naturalistic face recognition ability: it commonly fails to diagnose prosopagnosia, and tends to tap only the ability to match features such as the eyebrows [2], [50]. In the present study, we instead assessed face identity recognition with the Cambridge Face Memory Test (CFMT [2]), in which participants learn six faces, each in three viewpoints, to encourage face learning rather than simply image matching. The CFMT is a valid test of novel face learning (e.g., it diagnoses prosopagnosia well, and shows a large face inversion effect; see [1], [2]), has high reliability (typically.89-.90; see [7]), and is sensitive to individual differences in facial identity recognition (see [1], [2]). This allowed us to re-examine the question of whether there is a significant correlation between face identity (CFMT) and emotion-matching, as would be predicted by the model of Calder and Young [47], and how this compares in size to the correlation with the emotion-labelling.

### The Present Study

Each participant completed four core measures – face emotion-matching, face emotion-labelling, voice emotion labelling and face identity recognition (CFMT). In addition each participant completed two additional tasks. The Emotion Hexagon [17] is a test that is often used to assess facial expression recognition (4,170 results in a Google Scholar search September 24, 2012), and was included to provide an additional estimate of facial emotion-labelling from a well-established test. Cattell’s Culture Fair Intelligence Test (CFIT [51]) is a measure of non-verbal IQ, and was included so that we could examine divergent validity (i.e., demonstrate that our new tests were not tapping only general cognitive abilities). Only a few studies with typical adults appear to have examined the relationship between IQ (or proxies such as academic achievement or verbal ability) and variables we include in our study. Absent or low correlations are typically seen between IQ/general abilities and facial *expression* recognition (e.g., [15], [43]) and facial *identity* recognition (e.g., [7], [8]). For *vocal* emotion recognition, one study showed a small, albeit significant (with very large N), relationship with IQ (*r = *.18, *N* = 1,311) [43].

## Methods

### Ethics Statement

The ANU Human Research Ethics Committee approved the conduct of the project and each participant provided informed written consent.

### Participants

For the main study (all faces upright) data from 80 adults of European descent (51 female) aged from 18 to 49 years (*M = *23.16, *SD = *5.25) were analysed (nine individuals were excluded prior to analysis; three because they reported having experienced a significant head injury and six because they reported having a current clinical diagnosis of a mood or anxiety disorder, or another brain-related developmental disorder). The majority of participants were undergraduate students, recruited from either a third year cognitive psychology course at the Australian National University or via flyers posted around campus. On the CFIT, mean age-adjusted standard score was 122.56 (SD = 12.27), indicating that the sample displayed, on average, higher IQs than the general population. Participants were reimbursed $20, received course credit, or completed the experiment as a course requirement.

For the inverted-orientation validity test, a different group of 18 participants (15 females; aged 17 to 23 years M = 18.72, SD = 1.49), selected from the same population, were tested on the two main tasks.

### Design

Upright-orientation participants completed six tasks, in the following order: (i) the emotion-matching task developed as part of this project, (ii) the emotion-labelling task developed as part of this project, (iii) a vocal emotion labelling task [44], (iv) the Emotion Hexagon [17], (v) the standard CFMT [2], and (vi) the paper and pencil CFIT [51]. The first four tasks were controlled by SuperLab 4 (Cedrus Corp.) and were presented on either an iMac or a Dell PC, with image size adjusted to be equivalent regardless differences in the size of the monitor.

Because mean accuracy levels can often be close to ceiling when prototypical facial expressions are presented for an unlimited duration (see Table 1), in both of our new tasks we restricted the presentation time (but note that the duration was much longer than that for a micro-expression).

For the inverted orientation, we required data only for the two new tasks. Inverted-orientation participants were tested on exactly the same sequence of events as the upright-orientation participants up to the end of (ii) above. The only change was that all face stimuli in the emotion-matching and emotion-labelling tasks were rotated by 180 degrees in the picture plane.

### Tasks

#### 1. Emotion-matching task

The emotion-matching task involved the simultaneous presentation of the faces of three different individuals, with two expressing the same emotion and the third, a different ‘odd-one-out’ emotion. Participants were asked to identify which face displayed the ‘odd-one-out’ emotion.

##### Stimuli

Full-colour images of individuals of European descent displaying the six basic facial emotions (happy, sad, angry, surprised, disgusted and fearful) were selected from the Karolinska Directed Emotional Faces database (KDEF [20]). Ratings by Calvo and Lundqvist [52] and Goeleven, De Raedt, Leyman, and Verschuere [53] were used to select 144 target (or ‘odd’) faces, 24 from each emotion category (happy, angry, disgusted, sad, fearful, or surprised), of which eight faces displayed a full-face pose, eight a left facing three-quarter pose and eight a right facing three -quarter pose. A set of 288 distractor faces was also selected from the KDEF (see Table S1 for list of faces used). Each face was enclosed in an elliptical grey oval that excluded the hair but clearly showed the facial expression. Any blemishes judged as potentially distracting, such as moles or spots, were removed with Adobe Photoshop.

Each target (or ‘odd’) face was displayed in a triad with two distractor faces so that the expressions displayed by the target and distractor were maximally confusable, as per the Emotion Hexagon (i.e., happiness–surprise; surprise –fear; fear–sadness; sadness–disgust; disgust–anger; anger–happiness [17]). On half of the trials, a target was paired with two distractors displaying one of the confusable emotions (for example, a disgusted target with angry distractors), and on the other trials it was paired with two distractors displaying the other confusable emotion (for example, a disgusted target with sad distractors). To encourage processing of the facial expressions rather than simply low-level features, the target and distractor images were matched on low-level features. For example, open-mouthed happy expressions were matched with open-mouthed surprised distractors, so that participants could not simply match open vs. closed mouths with no reference to the expressions. The faces in each triad were different individuals but they were all the same sex and displayed the same viewpoint (full-face, left-facing three-quarter or right-facing three-quarter) (see Figure 1 for examples). The position of the target and distractors (left, right, or middle) on each trial was initially allocated randomly, with these positions then maintained for every participant. Based on a viewing distance of 50 cm, each face was approximately 9°×6.5°, and there was 5.5°between each face in the triad.

##### Procedure

Each trial began with the word “READY” presented in the centre of a grey screen for 500 ms, followed by a triad of three faces. Participants were asked to use the 1, 2 and 3 keys on the computer keyboard to indicate which of the facial expressions differed from the other two. Participants were able to respond while the faces were presented (4,500 ms) and for an additional time window (7,000 ms) after the faces had been erased. Participants were instructed to respond as quickly and accurately as possible.

Presentation order for the 144 trials was initially randomized, and this same presentation order was then administered to all participants. A rest break was provided after each block of 30 trials. Eight practice trials were completed initially. The task took approximately 15 minutes to complete. An accuracy score was calculated from the total correct responses, where the maximum was score was 144 (chance = 48).

#### 2. Emotion-labelling task

In this task, a single face displaying one of six basic emotions was shown and participants were asked to specify which emotion label was most appropriate.

##### Stimuli

The target faces were the 144 target faces from the Emotion-Matching task (24 from each emotion category; eight full-face pose, eight left facing three-quarter pose, eight right facing three-quarter pose) presented on a grey background. At a distance of 50 cm, the faces were approximately 8.5°×5.5°.

##### Procedure

Each trial consisted of the word “READY” presented in the centre of the screen for 500 ms, followed by the target face. Participants were required to use the computer mouse to select the appropriate emotion label from the six alternatives listed underneath the face (from left to right: Angry, Disgusted, Fearful, Happy, Sad, or Surprised). Participants were able to respond while the faces were presented (1,000 ms) and for an additional time window (7,000 ms) after the faces had been erased but in which the labels remained on the screen. Participants were asked to respond as quickly and accurately as possible.

The presentation order for all 144 items was initially randomized, and this order was used for all participants (which is important given that facial expression categorisation can be affected by the expression of the face previously seen [54]). The task took approximately ten minutes to complete, with the opportunity for a rest break after every 30 trials. Participants completed four practice trials prior to the experimental trials. An accuracy score was calculated from the total correct responses, where the maximum was score was 144 (chance = 24).

#### 3. Vocal emotion-labelling task [44]

In this task, vocal emotions were presented corresponding to one of five basic emotions (sadness, anger, fear, disgust and happiness [surprise was not part of the set]) and participants were asked to indicate which emotion label was most appropriate.

##### Stimuli

The stimuli, developed by Calder and colleagues [44], consisted of 50 audio recordings of actors repeating random strings of digits (e.g. ‘9, 5, 1, 2, 7’) in a tone that represented one specific emotion. Ten exemplars were presented for each of the five emotion categories, with each recording of 2000-3000 ms duration. Five practice stimuli were also initially presented.

##### Procedure

Participants listened to the vocally presented emotions via headphones, with the sound level individually adjusted by the participant. Participants were instructed to use the mouse button to click on the emotion label (Angry, Disgusted, Fearful, Happy, or Sad) that best described the emotion expressed by the tone of voice. Participants were able to make a selection during the presentation of the vocal stimuli or for up to 6,000 ms afterwards. The presentation order of the 50 stimuli was initially randomised and then presented in this fixed order across participants. Five practice trials were initially completed to familiarise participants with the task. Overall, this task took approximately six minutes to complete. An accuracy score was calculated from the total correct responses, where the maximum score was 50 (chance = 10).

#### 4. Emotion hexagon [17]

This task consists of greyscale photographs of one individual displaying six basic emotions, which were blended together on the basis of a confusion matrix (happiness–surprise; surprise –fear; fear–sadness; sadness–disgust; disgust–anger; anger–happiness) in five steps (90∶10, e.g., 90% happiness: 10% surprise; 70∶30; 50∶50; 30∶70; and 10∶90). Participants viewed each blend for 5,000 ms and were given unlimited time to use the computer mouse to select the label (angry, disgusted, fearful, happy, sad, surprised) which best described the emotion displayed. Participants completed one practice and five experimental blocks, with each block containing the same 30 images. The order of presentation within each block was randomised for each participant. The task took approximately 10 minutes to complete. As per the manual, data from trials in which the two emotions were expressed at an equal intensity (50∶50; 6 trials per block) were not included in analyses, so that total accuracy was based on the total correct from 120 trials (chance = 20).

#### 5. Cambridge Face Memory Test (CFMT) [2]

Participants completed the upright version of CFMT, a test of face learning and recognition consisting of three stages of increasing difficulty, following the standard instructions. In the first stage, participants learn six target faces in three views and then select which was the target face from two simultaneously matched distractor faces. Identifying the target faces becomes more difficult in subsequent stages, with lighting conditions and viewing angles changed in stage two and coloured noise added to the images in stage three. A total score out of 72 is obtained by summing across the three stages (chance = 24). This task takes approximately 10 minutes to complete. The CFMT displays high reliability (Cronbach’s α, = .89), validity, and sufficient range to measure individual differences [1].

#### 6. Cattell Culture-Fair Intelligence Test (CFIT) [51]

The CFIT is generally regarded as a gauge of fluid intelligence [55]–[57] and uses a nonverbal format [56], [57]. The CFIT, Scale 3, Form A, consists of 50 geometric items, divided into four subtests (Series, Classifications, Matrices, Topology), with participants required to select one option from several alternatives on the basis of a particular principle, e.g. similarity, continuation of a geometric pattern, etc. Scale 3 is designed for use with “individuals considered… higher in ability level” (as suitable for our university population) and has a Cronbach’s α of.74 ([51], p.7). The CFIT was administered as per the standard instructions in the test manual, which included practice questions, and took approximately 20 minutes.

## Results

### Validity, Reliability, and Psychometrics for our New Expression Matching and Labelling Tasks

We initially examined results for the full 144 items tested in each task. Performance on both tests, with average accuracy of 74%, was well away from both floor and ceiling (see Results S1 for more details), as desired for tests designed to assess individual differences in the normal range. Reliability, in terms of internal consistency, was assessed using Cronbach’s alpha, and was initially.74 for the all-items emotion-matching task and.64 for the initial all-items emotion-labelling task.

We then selected final 100-item versions of each test from the initial 144 items, with the aim of improving both reliability and construct validity. Validity was examined first. Target faces (i.e., the faces used in the labelling test, and as the odd-expression-out targets in the matching test) were considered to have low validity and removed if the intended emotional expression was not the emotion most frequently selected by participants in the labelling task (e.g., if the original KDEF database listed the face as “Fearful” but in fact more participants labelled it as “Surprised” than “Fearful”). Twenty-three targets (1 angry, 2 sad, 2 surprised, 8 disgusted and 10 fearful) were removed (from both tests) on this basis.

The individual contribution of each of the remaining 121 items to internal consistency was then estimated by calculating the value of Cronbach’s alpha if that item were to be excluded from the test. Items were excluded until (a) reliability plateaued, and (b) both tasks still contained the same target items. Table S1 lists all items included in the original test and those included in the 100-item versions. For each test separately, we also progressively removed target faces until no further improvement in reliability emerged and the highest level of reliability was obtained (See Table S1 for details of the resulting 65-items for the matching task and 48 items for the labelling task and Results S1 for details of their distributions).

The reduction from 144 items to the final 100 items led to a small improvement in reliability on emotion-matching (α = .77) and a substantial improvement for emotion-labelling (α = .76). The improvement was particularly large for male participants in both tasks (from.55 to.71 in the emotion-matching task and from.54 to.75 in the emotion-labelling task), and with the 100-item tests reliability was now reasonably comparable across males (.71,.75, matching and labelling respectively) and females (.79,.77).

**Figure 2:**
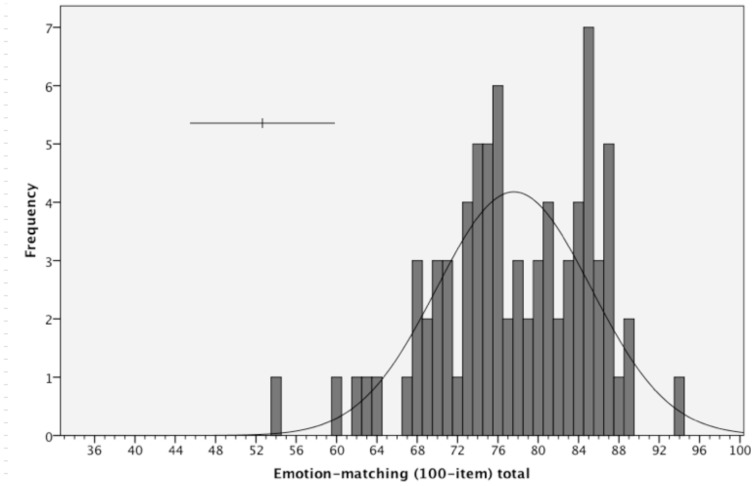
Frequency distribution for scores on the 100-item Emotion-matching task (chance performance = 33). Error bars indicate 95% confidence interval of individual scores based on task reliability.

**Figure 3:**
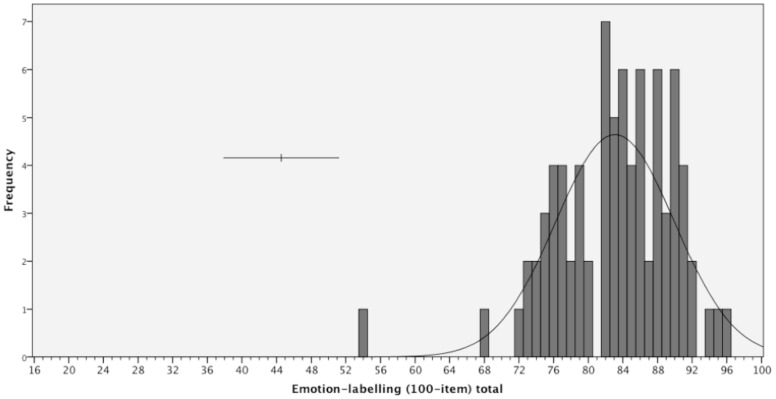
Frequency distribution for scores on the 100-item Emotion-labelling task (chance performance = 8). Error bars indicate 95% confidence interval of individual scores based on task reliability.

The frequency distributions of scores on the 100-item emotion-matching and -labelling tasks are presented in Figures 2 and 3 respectively. For the 100-item emotion-*matching* task, the distribution was not significantly different from a normal distribution (Shapiro-Wilks tests revealed *W = *.97, *df* = 80, *p* = .09) and not significantly skewed (skew = −.49, SE = .27, z = 1.81, p = .08). Performance was well away from both floor and ceiling (Matching: 77.59/100, SD = 7.64, chance = 33) (Table 2). On the 100-item emotion-*labelling* task, performance was also neither at floor or ceiling (Labelling: 83.10/100, SD = 6.87, chance = 17; Table 2). When all participants were included, the data were not normally distributed (*W = *.94, *df* = 80, *p* = .001) with significant negative skew (skew = −1.02, SE = .27, z = 3.77, p<.001). This appeared to be driven by the presence of an outlier that was 4.23 SD below the mean; without this participant, the data were not significantly different from a normal distribution (*W = *.98, *df* = 79, *p* = .26) and were not significantly skewed (skew = −.24, SE = .27, z = .89, p = .19).

**Table 2:** Descriptive statistics for each task, including possible and observed range, Mean (Standard Deviation) and Cronbach’s alpha (α).

Task	Task Range		Total (N = 80)		Female (n = 51)			Male (n = 29)	
	Chance – Max	Observed	M (SD)	α		M (SD)	α	M (SD)	α
Emotion-matching task (100-item)	33–100%	54–94%	77.59 (7.64)	0.77		78.76 (7.86)	0.79	75.52 (6.88)	0.71
Emotion-labelling task (100-item)	16.67–100%	54–96%	83.10 (6.87)	0.76		84.06 (6.81)	0.77	81.41 (6.77)	0.75
Emotion Hexagon [17]	16.67–100%	51.67–100%	88.92 (9.38)	0.92^c^					
Vocal emotion-labelling task	20–100%	56–96 %	76.13 (9.25)^a^	0.69					
Cambridge Face Memory Test (CFMT) [2]	33–100%	43.06–97.22%	77.49 (13.33)^b^	0.90					
Cattell’s Culture Fair Intelligence Test(CFIT) [51]	55–183	94–152	122.56 (12.27)	0.74^c^					

a N = 79

b N = 78

c Obtained from Test Manual

There were no significant sex differences on the 100-item matching (female *M* = 78.76, *SD* = 7.86; male *M* = 75.52, *SD* = 6.88, *t*(78) = 1.86, *p* = .07) or labelling (female *M* = 84.06, *SD* = 6.81; male *M* = 81.41, *SD* = 6.77), *t*(78) = 1.67, *p* = .10) tasks. There were no correlations between age of the participant and either the matching or labelling tasks (*r’s* <.15, *p’s* >.24), as expected given the restricted age range (18–49 years). Additional participants have been tested with only the 100 items (rather than taking the 100 items as a subset of the 144 items version); the distributions and reliability are similar to those reported here (Results S2).

The final 100-item versions resulted in unequal numbers of each of facial expression (22 happy targets, 20 angry, 17 sad, 15 surprised, 14 disgusted, 12 fearful). This was unavoidable given that actors’ posing of expressions tends to be most convincing and unambiguous for happy and angry, while expressions such as fear tend to be posed less convincingly or less unambiguously. Given that the aim of our tests was to provide a tool for measuring *overall* emotion recognition ability, rather than the ability to recognise specific individual emotions, we felt that any potential disadvantages of the unequal numbers of each expression were offset by the improved validity and reliability. Concerning reliability for the recognition of *specific* facial emotions, the tests are moderately reliable in measuring the recognition of some specific emotions (see Results S3). For the 100-item emotion-matching task, reliability varies from 0.21 (fear) to 0.59 (happiness), and for the 100-item emotion-labelling task reliability ranges from 0.52 (happiness) to 0.68 (anger). Reliability for labelling expressions in our task (using naturalistic photographs of multiple different people) appears to be only moderately lower than split-half reliabilities reported for the Emotion Hexagon, which involves labelling morphed expressions displayed by the same person (range from.18 for happiness to.88 for fear, n = 20 trials for each expression; see Method for more details on the task) [17].

We now consider three other pieces of data relevant to determining task validity. First, neither of our two tests showed any correlation with IQ, indicating divergent validity (specifically, that our tasks do not measure merely general cognitive abilities). Our nonverbal IQ (CFIT) data were not significantly different from a normal distribution (*W = *.99, *df* = 80, *p* = .54), not significantly skewed (skew = −.05, SE = .27, z = .20, p = .39), and had no ceiling or floor effects (Table 2). Despite this good range, there was no relationship between the CFIT and either the 100-item emotion-matching task (*r* = .07, *p* = .56) or labelling task (*r* = .04, *p* = .74, *rho* = −.02, *p* = .88; note the nonparametric Spearman’s rho is reported where the outlier participant is included making labelling distributions non-normal).

Second, we examined convergent validity, to the extent this was possible, by examining the correlation between our new labelling task and the previously-developed labelling task we included, namely the Emotion Hexagon. Despite the Hexagon’s poor range for observing typical-population correlations (skewed distribution, W = .86, df = 80, p<.001, skew = −1.49, SE = .27, z = 5.54, p<.001; with very high mean M = 88.92%, SD = 9.38, see Table 2; and ceiling effects with some participants 100% accurate), we were able to observe a significant correlation between our new labelling task and this previous labelling task (*r* = .26, *p* = .02; *rho* = .23, *p* = .04).

Third, validity was further established by demonstrating significant inversion effects on each of our new tests (i.e. superior performance with upright than inverted faces). The presence of a strong inversion effect argues that participants were using normal facial expression recognition processes (which are disrupted by face inversion) and were not simply using low-level cues to perform the tasks. In the 100-item versions, highly significant inversion effects were evident on both our new tasks: Emotion-matching (upright: *M* = 77.59, *SD* = 7.64; inverted: *M* = 68.28, *SD* = 6.88; *t*(96) = 4.75, *p*<.001) and Emotion-labelling (upright: *M* = 83.10, *SD* = 6.87; inverted: *M* = 67.50, *SD* = 9.84; *t*(96) = 7.99, *p*<.001). The inversion effect magnitude was greater in the 100-item versions (matching: 9.31%, labelling: 15.60%) than the 144-item versions (matching: 7.78%, labelling: 12.91%), although the 144-item versions did also show clear inversion effects (emotion-matching upright: *M* = 74.10, *SD* = 6.34; inverted: *M* = 66.32, *SD* = 7.56; *t*(96) = 4.54, p<.001; emotion-labelling upright: *M* = 74.02, *SD* = 5.08; inverted: *M* = 61.11, *SD* = 8.64; *t*(96) = 6.10, p<.001). These results argue that both versions validly measure facial expression processing, but the 100-item versions have the highest validity (consistent with the removal of ambiguous-expression low-validity individual items in this version).

Overall, our new expression tasks in the 100-item version meet our requirements of (a) the same target items in the matching and labelling versions, (b) good range for testing individual differences in the typical population (as opposed to a range suitable only for categorical clinical versus unimpaired diagnosis, (c) high internal reliability (.77 for matching and.76 for labelling), and (d) demonstrated construct validity.

### Using our New Tasks to Address Theoretical Questions

We now use our new tasks to examine our three theoretical questions. All analyses were conducted using the 100-item emotion-matching and –labelling scores (Table B in Results S1 contains analogous analyses for the longer and shorter versions of the tasks, which show very similar patterns of results).

Before proceeding, an important general point is that our results so far confirm that there *are* genuine individual differences in facial emotion recognition ability in our non-clinically selected population; that is, we are not all “face emotion experts”. The evidence comes from the Cronbach’s alpha estimates of.77 and.76 (Table 2). These values imply that approximately three-quarters of the total variance associated with the overall scores was systematic. Thus, the high Cronbach’s alpha values indicate the presence of reliable individual differences in task performance. If the differences in individuals’ scores represented simply measurement error (i.e., noise), then the internal reliability of a task would be zero. Note that we were interested in contrasting emotion perception (matching) with recognition (labelling) and it was difficult to conceive of how a matching task could be created using dynamic displays while avoiding low-level differences (e.g., teeth display) that would make the task trivially easy. However, we note that the proportion of variance explained in emotion recognition by individual differences might differ if dynamic rather than static faces were used, and furthermore, that the answers to the theoretical questions addressed here may vary if dynamic faces were used.

To address our theoretical questions, we report parametric statistics (e.g., Pearson’s *r*), but also non-parametric statistics (e.g., Spearman’s *rho)* in cases where distributions were significantly different from normal and skewed. All participants were included: the outlying individual on labelling also scored poorly on the other emotion tasks (emotion-matching task z = −1.39; vocal emotion-labelling z = −2.18,) but not the non-emotion tasks (CFIT z = −.78; CFMT z = 0.02), so we opted to retain this person’s data as reflecting actual individual differences.

Our analyses compare each correlation between two tests to the theoretical upper bound of the correlation that could be obtained, calculated as the square root of the product of their reliabilities [26]. Comparison to upper bound is important because the maximum possible correlation is often well below 1, and thus complete overlap in processing between two tasks predicts an observed correlation of upper bound, not 1.

#### 1. Relationship between emotion-matching and –labeling

**Figure 4:**
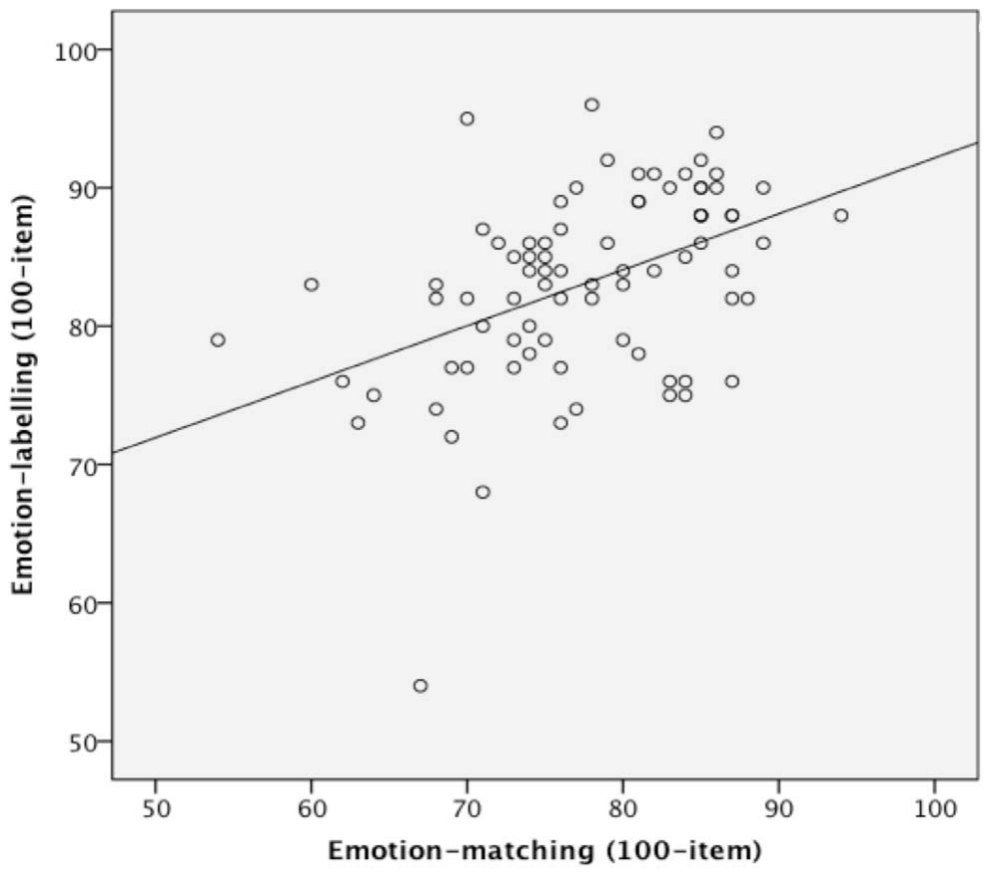
Scatterplot of scores on the 100-item emotion-matching and -labelling tasks.

The correlation between the emotion-matching and emotion-labelling tasks was positive, indicating that participants with better emotion labelling tended to also have better emotion labelling, and moderate-to-large in size (*r* = .45; *rho* = .47, *p’s* <.001, Figure 4). At the same time, it was noticeably below upper bound (r_upper_ = .76). Together, these results argue that, theoretically, face emotion matching and face emotion labelling tap partially overlapping processing stages (significant positive correlation), but are not completely identical constructs tapping fully overlapping processes (correlation below upper bound). Note that the matching-labelling correlation was not significant when the Emotion Hexagon was used as the labelling task (r = .15, p = .17 [r_upper_ = .84]; rho = .21, p = .06); however we do not consider this finding of theoretical importance due to the poor range on the Hexagon labelling task compared to our new task.

The correlation between the matching and labelling tasks was maintained at similar magnitude (*pr(*76) = .41, *p*<.0001) when individual differences in face *identity* recognition performance (CFMT scores) were included in a partial correlation. This argues that the overlapping processes tapped by the two emotion tasks are specific to emotion, and are not general face recognition processes. Moreover, the correlation between matching and labelling was also maintained when *vocal* emotion labelling scores were included in a partial correlation, *pr*(76) = .44, *p*<.0001. This indicates that the processes common to emotion-matching and -labelling represent ‘face’ emotion processing, not just ‘general’ emotion processing from any modality.

Finally, we checked that the strength of the relationship between the emotion-matching and -labelling tasks was not simply driven by the use of the same stimuli across tasks. This was confirmed by splitting the data from each task into two halves, with the target expressions in one half of the matching task different to those in one half of the labelling task, and then correlating these two halves (i.e., Match 1 with Label 2; Match 2 with Label 1). The average of the two correlations was.57 with a Spearman-Brown correction for list-length (.39 without correction), which is not smaller than the original r = .45, and thus clearly demonstrates that the strength of the correlation between the emotion-matching and -labelling tasks was not simply due to the same stimuli being used across the tasks.

**Figure 5:**
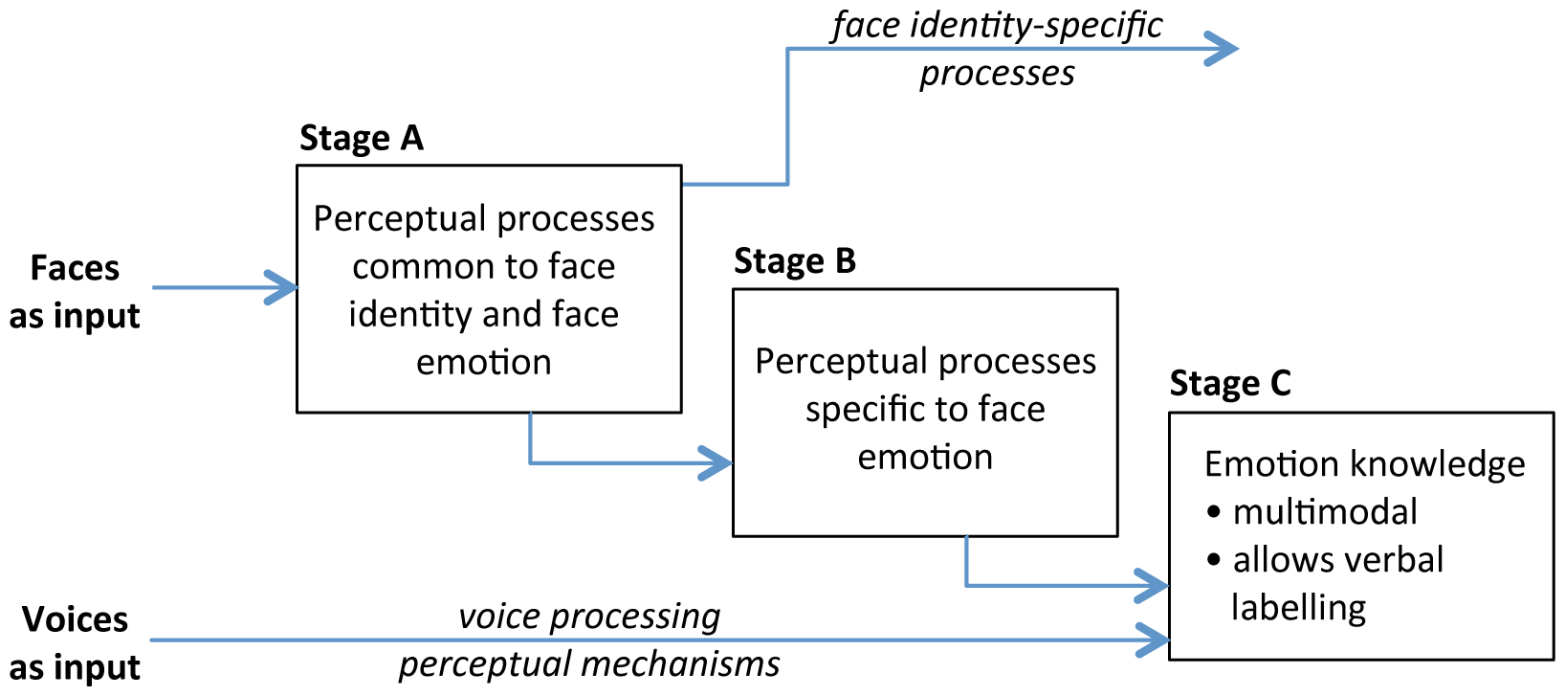
A structure of face and emotion processing mechanisms that is consistent with the observed pattern of correlations. (Note that the arrows are likely to be bi-directional indicating both feed-forward processing and top-down feedback but as our data does not address this they are represented as simply unidirectional).

Overall, these results argue that (a) face emotion-matching and face emotion-labelling tap partially overlapping and partially distinct processes, and that (b) there are overlapping processes between the two emotion tasks that are independent from processes tapped by face identity recognition and by vocal emotion recognition. Theoretically, the most plausible interpretation is then that there are high-level perceptual processes that are specific to face emotion, and these perceptual processes contribute to both face matching and face labelling (Figure 5 - Stage B) and that there are also post-perceptual additional processes that contribute specifically to labelling face emotions (Figure 5 - Stage C).

#### 2. Relationship between recognising emotion from faces and voices

For the vocal emotion-labelling task, the data from one participant was deleted as inspection revealed their poor performance (*M* = 44%) was most likely to be due to inattention to the task, with ‘happy’ used in 62% of responses. The data from the remaining 79 participants were not normally distributed (*W = *.97, *df* = 79, *p* = .04), although there was no significant skewness (skew = −.31, SE = .27, z = 1.14, p = .21). Average accuracy on the task was neither at ceiling or floor (*M* = 76.13%, *SD* = 9.25; range = 56–96%) and Cronbach’s alpha was acceptable at.69 (Table 2).

Of interest, vocal emotion labelling performance was associated with IQ. The correlation with CFIT scores was *r* = .27, *p* = .02 [r_upper_ = .71]; *rho* = .23, *p* = .04.

**Figure 6:**
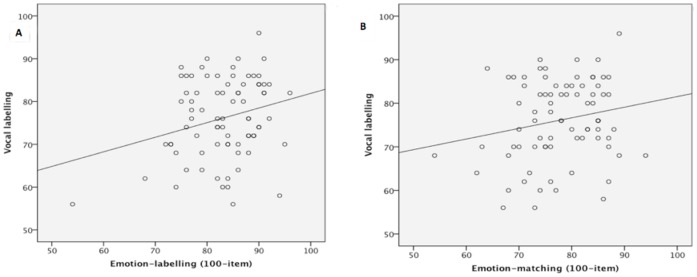
Scatterplot of scores on the vocal labelling task and (a) 100-item emotion-labelling task, and (b) 100-item emotion-matching task.

Performance on the *vocal* emotion-labelling task was associated with that on our *face* emotion-labelling task with parametric analyses (*r* = .26, *p* = .02 [r_upper_ = .72]) but only approached significance with non-parametric analyses (*rho* = .19, *p* = .09) (Figure 6a). However, two additional results reinforce the finding of a cross-modal association between labelling faces and voices. First, the relationship between vocal and face labelling was still apparent when partial correlations were conducted to control for IQ (CFIT scores, *pr* = .25, *p* = .03). Second, we also found that the ability to label vocal emotions was associated with the ability to label facial expressions in the Emotion Hexagon task [17], *r* = .25, *p* = .02 [r_upper_ = .80]; *rho* = .25, *p* = .02 (despite the poor range on this task), which also remained evident when the influence of CFIT scores were partialled out, *pr* = .23, *p* = .05.

These results indicate a cross-modal association, the simplest interpretation of which is that this reflects a post-perceptual emotion *labelling* stage that is accessed regardless of the format in which the emotion is conveyed (i.e., via the face, or the voice or, presumably, other means such as body posture) (Figure 5– Stage C). Supporting this interpretation, there was no significant correlation between the face emotion-*matching* task, which we suggest taps only the face emotion *perception* stage (Stage B) but not the subsequent labelling stage (Stage C) and the vocal emotion-labelling task (*r* = .20, *p* = .08 [r_upper_ = .73]; *rho* = .16, *p* = .16, Figure 6b. There was also no relationship when CFIT scores were included as a covariate, *pr* = .15, *p* = .20).

#### 3. Relationship between facial identity and facial expression recognition

The face identity recognition (CFMT) data were not normally distributed (*W* = .95, *df* = 79, *p = *.003) and showed small but significant skew (skew = .−57, SE = .27, z = 2.08, p = .05). Performance was neither at ceiling nor floor (M = 77.49%, SD = 13.33, range = 43.06–97.22%) and Cronbach’s alpha for this sample was.90 (Table 2). As expected face identity recognition (CFMT) performance was not associated with IQ (CFIT ability, *r* = −.01, *p* = .96 [r_upper_ = .82]; *rho* = −.04, *p* = .74).

**Figure 7:**
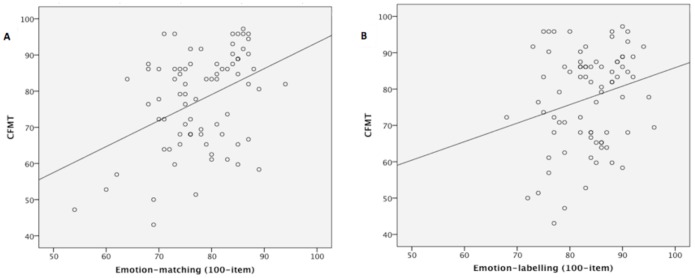
Scatterplot of scores on the CFMT and (a) 100-item emotion-matching task, and (b) 100-item emotion-labelling task.

There was a moderate positive association between the ability to recognise facial identity and emotion-*matching* (*r* = .40, *p*<.001 [r_upper_ = .83]; *rho* = .34, *p* = .002) (Figure 7a), consistent with the Calder and Young’s [47] theory that there some early stage/s of perceptual face processing are shared in common by face identity and face emotion processing (Figure 5– Stage A). Consistent with this interpretation, the correlation between the CFMT face identity task, which requires perception but does not include any labelling requirement, and emotion-*labelling* was weaker: it only approached significance using our new test of emotion labelling (*r* = .20, *p* = .08 [r_upper_ = .83]; *rho* = .19, *p* = .10 (Figure 7b), and was significant but small with the Emotion Hexagon labelling task (*r* = .27, *p* = .02 [r_upper_ = .91]; *rho* = .27, *p* = .02).

## Discussion

In the present study, we developed new tests of emotion-matching and emotion-labelling. Using these tests, we were able to demonstrate that there are real, measurable individual differences in emotion recognition ability in the typical adult population. We also used these tests to investigate three important theoretical questions about the perceptual structure of face processing, which have been the focus of little previous research.

### Valid and Reliable New Tests of Face Emotion-matching and -labelling

We developed two new tests that displayed good validity and reliability and were not constrained by floor or ceiling effects. As evidence for validity: we carefully selected items to remove any with ‘poor’ expressions where most observers did not agree with the label assigned by the database to the expression; we demonstrated divergence from general nonverbal intelligence; we demonstrated convergence between our labelling task and a previous labelling task (the Emotion Hexagon); and we demonstrated highly significant inversion effects. Regarding reliability, in the 100-item versions internal consistency (Cronbach’s alpha) was.77 for face emotion matching and.76 for face emotion labelling. These values are adequate for tests designed to examine correlations with moderate sample sizes. Concerning score range, the lack of ceiling and floor effects (consistent with normal not skewed distributions) indicate that our tests are suitable for individual differences analyses in the typical population, rather than merely for binary classification of clinical versus nonclinical status.

Importantly, our results argue that our two tests do not assess identical constructs. Although the tests correlated together (at *r* = .45), this correlation was well below upper bound. Moreover, one test correlated with *vocal* emotion-labelling while the other did not. This implies that, where researchers wish to use our tests in future, it is important for those researchers to decide which test to use based on what theoretical construct/s they wish to measure. The theoretical understanding developed from the second aim of our article will facilitate this choice.

### Theoretical Implications of Our Correlations

**Figure 8:**
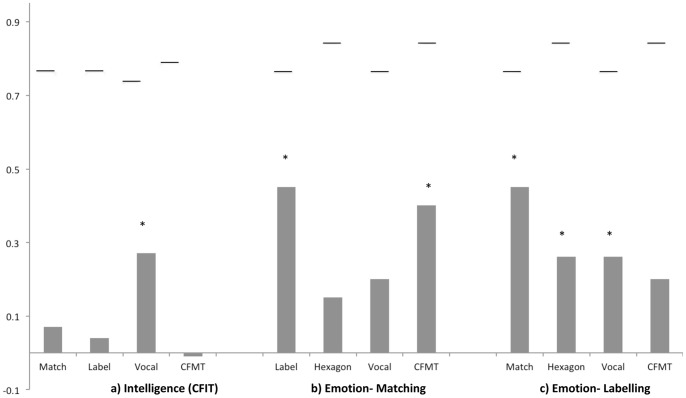
Correlations between the Emotion-Matching task (Match), Emotion-labelling task (Label), vocal labelling task (Vocal), and the Cambridge Face Memory Test (CFMT) and a) IQ as measured with the CFIT (Cattell’s Culture Fair Intelligence Test), b) Emotion-Matching task and c) Emotion-Labelling task. Pearson correlations significant at the 0.05 level are signified with a *. Horizontal lines above each bar indicate the upper bound for the correlation based on internal reliability. Note that all correlations with the Emotion Hexagon may be further limited by the low score range (ceiling effect) on that task.

We summarise all the observed correlations in Figure 8, and Figure 5 shows a structural model of face and emotion processing mechanisms that is consistent with our overall pattern of significant and non-significant correlations.

First, consider Stage A in Figure 5. As evidence for this stage, our results support the existence of mechanisms that are common to memory for face identity, matching images of facial emotions without applying an explicit label to the emotion, and labelling the emotion in those facial images (i.e. all correlate together). For this pattern of results, a parsimonious explanation is that there is an initial stage of high-level face processing that is common to both face identity and face emotion processing. This conclusion is in agreement with the theoretical ideas of Calder & Young [47] (also [48], [58]) but not consistent with earlier models in which the split between the processing of identity and expression information is quite early (before the stage of view-independent ‘structural descriptions’ in the cognitive model of Bruce and Young [45], and before processing in the lateral fusiform gyrus (identity) and superior temporal sulcus (expression) in the anatomical model of Haxby et al. [46]).

The existence of Stage A is also supported by other findings in the literature. A correlation between emotion-labelling and face identity *perception* (as opposed to our *memory* task) has been reported in previous studies (e.g., [12], [13]; both using the Benton Facial Recognition Test). Also, recent fMRI evidence indicates that some areas in the ventral temporal cortex that are responsive to faces are responsive to both changeable (e.g., expression) *and* unchangeable (e.g., identity) dimensions, while some subregions are specialised for either changeable *or* unchangeable dimensions [59]. Behaviourally, results also suggest at least one candidate perceptual mechanism might be common to both face identity and face emotion, namely holistic processing, in which the features of a face are perceptually integrated (e.g., [60], [61]). In individuals with congenital or developmental prosopagnosia, who have difficulty recognising the identity of familiar faces in everyday life, holistic coding is impaired for both face identity [62], [63] and face expression [63].

Overall, together with recent computational, neuropsychological and neuroimaging research (reviewed in [48]), we argue that there is now good evidence for an initial high-level perception stage in face processing which contributes to identity and emotion processing equally. Individual differences studies using our new tests have the potential to further contribute to understanding of the mechanism/s involved in this stage of processing. The strength of holistic processing is associated with the ability to recognise facial identity in typical individuals [64], but a relationship between holistic coding for identity and expression has yet to be demonstrated in typical individuals (as opposed to those with prosopagnosia). We are currently investigating this question by using holistic processing measures in conjunction with the tasks designed in this study. Future correlational studies can also investigate other perceptual mechanisms to determine whether these are, or are not, common to the processing of identity and expression (e.g., strength of face-space coding, and strength of part-based face coding, both of which correlate with face identity recognition ability [65], [66]). It would also be of interest to examine whether individual differences in ability could be modified by prior perceptual context, such as viewing one’s own facial expressions prior to completing the tests (c.f., [67], who observed that viewing one’s own, as compared to another person’s, facial expressions speeded subsequent discrimination between facial expressions).

We next consider Stage B in Figure 5, namely a stage of process/es specific to face emotion, which is engaged specifically in perception of facial emotion but without including broader semantic knowledge about emotions per se (such as the verbal labels applied to emotions). The existence of this stage, and its separation from both Stage A and Stage C, is consistent with the following findings. First, we demonstrated a moderate to large correlation between our two facial emotion tasks (matching and labelling). It also important to note that the size of our correlation, derived from tasks containing images from the KDEF database [20] is numerically similar to that reported by Addington and Addington [12], who used tasks with different parameters and presentation durations and a different stimulus set, the Pictures of Facial Affect [68]. Second, the correlation between our two face emotion tasks was below upper bound, consistent with a view in which perceptual face emotion processes (Stage B) are tapped by both matching and labelling, but that the labelling task additionally taps further, later, processes (Stage C). Third, the strength of the correlation between our two emotion tasks was not reduced when either face identity (Stage A) or vocal emotion labelling (Stage C) were included as covariates, arguing Stage B is independent of both other stages.

These ideas are also consistent with neuroimaging results. Hariri, Bookheimer, and Mazziotta [69] compared brain activation for matching and labelling tasks, and found that both activated a face-selective area of the posterior fusiform gyrus known as the fusiform face area (FFA [70]), which is involved in the perception of both facial identity and facial expression (see review by Calder [48]), consistent with the existence of Stage B. They also report there are some differences, with greater amygdala activation during matching than labelling, but greater prefrontal cortex activation during labelling than matching [69], [71], consistent with processing in a separate Stage C that deals with labelling emotions.

Finally, we turn to Stage C in Figure 5, and the proposal that the labelling stage is multimodal. Evidence for Stage C is that vocal labelling correlated with face emotion *labelling,* both our newly developed task and the Emotion Hexagon (arguing for shared processes involved in labelling) but did not significantly correlate with face emotion *matching* (arguing for separation from Stage B). Our proposed separation between Stages B and C fits with current models of face and voice perception. For instance, Belin, Bestelmeyer, Latinus, and Watson [72] propose that structural information from voices and faces is initially processed independently (c.f., our Stage A), and then the affective components are processed both independently (c.f., our Stage B) and interactively in a multi-modal emotion processing system (c.f., our Stage C).

The correlation between face and vocal emotion tasks suggests Stage C involves emotion processing in general, rather than just facial expression or voice expression processing. This association between emotional face and voice processing may derive from processing in regions of the superior temporal cortex, regions that are consistently activated in response to facial expressions (see reviews by Calder & Young [47], Haxby et al. [46]) and which may contain “emotional voice areas” [73]. Alternatively, common processing of emotional information from faces and voices may occur in multimodal areas such as somatosensory cortices [74] or higher association areas, including frontal or posterior cingulate cortex, and subcortical areas such as the amygdala [75], [76].

However, there are two caveats to this interpretation of Stage C from our data. First, although there was an association between labelling emotion from voices and faces, the small size of the correlation appeared to be indicative of a fairly weak relationship between the two variables. One reason for only a small association between the ability to discriminate emotion from voices and faces could be that vocal characteristics may be more useful for appreciating levels of emotional *arousal* whereas facial expressions may be more likely to convey *valence* [77]. In many dimensional models of emotion (e.g., Circumplex Model [78]), arousal or the strength of the response to a stimulus, and valence or pleasantness, are typically viewed as orthogonal dimensions, that can be assessed independently (e.g. [79]) and may recruit different brain regions (e.g. [80]). Second, while the relationship between vocal emotion-labelling and facial emotion-matching was not significant, it was of a similar numerical size to the significant relationship between the vocal and facial labelling tasks (See Figure 8). It is possible that correlations between the vocal and facial labelling tasks were apparent simply because of the shared linguistic components, which are absent in the matching task. If so, it is possible that an association may have been found between a vocal emotion-matching task and a facial emotion-matching task, as in [13]. Further research is needed to evaluate these alternatives.

### Correlations with IQ and the Independence of Face “Module/s” from General Cognition

Finally, we also investigated whether there were associations between IQ and face and voice processing.

For faces, consistent with most previous studies, there was no significant association between IQ and facial emotion matching, facial emotion labelling, or facial identity recognition. This makes a strong case that face processing is a specific ability. For face *identity*, we also know from previous individual-differences studies of twins that this specificity extends to heritability; that is, face identity abilities are heritable independent of the heritability of IQ [7], [8]. Interesting open questions concern (a) whether face *emotion* ability is also heritable independent of IQ (a recent twin study of face emotion labelling found a heritable contribution to performance but did not measure IQ, plus the reliability of their expression recognition task was not reported [18]), and (b) whether face emotion and face identity abilities are heritable independently *from each other*, or whether the independence of heritability is only from IQ. Our new facial emotion-recognition tasks developed in this study provide suitable measures for future examination of these questions.

In contrast to our face tasks, we did observe an association between vocal emotion labelling and IQ, an association that has been reported at least once before (but with a much larger sample [43]). This might be explained by higher working memory demands for vocal than facial emotion recognition tasks (e.g., vocal information must be kept in mind while attending to visual labels [81]). The finding of a relationship between IQ and vocal emotion recognition indicates that studies investigating vocal emotion recognition, particularly those with an individual differences approach or employing special populations, would benefit from including measures of IQ.

### Conclusions

To summarise, this study detailed the development of two new tests, one for expression perception (an odd-man-out *matching* task) and one requiring explicit identification of the emotion (a *labelling* task), and demonstrated that the tasks are valid, reliable and that there are wide individual differences in the ability of the typical adult population to recognise facial expressions of emotion. Theoretically, our results using these tests supported a structure of face processing that contained three stages: high-level perceptual processes common to face identity and face emotion; high-level perceptual processes specific to face emotion; and an emotion knowledge stage that is multimodal in input and allows verbal labelling of the expressed emotion.

Given the validity, reliability, score range, and brevity (∼ 10 mins for 100 items) of our two new tests, we expect them to have wide applicability to future studies investigating facial expression processing in the typical adult population. We have given examples of potential future uses of our tests in the perception and cognition domain (e.g., examining the contribution of holistic processing to individual differences in expression recognition; examining heritability). Equally, our tests are also suitable for researchers interested in questions from the psychosocial domain, such as what personality or life-experience factors might be associated with individual variation in the important skill of face emotion processing (e.g., empathy, extroversion, maternal attachment style).

## Supporting Information

Results S1 **Analysis of other versions of the emotion-matching and emotion-labelling tasks (144, 65, and 48 item versions).**
(PDF)

Results S2 **Reliability for separate expressions in the 144-item and 100-item emotion-matching and –labelling tasks.**
(PDF)

Results S3 **Performance on 100-item matching task by different sample.**
(PDF)

Table S1 **KDEF stimuli used in the tests.** Left columns display the expression and KDEF label of faces selected for Emotion-matching and labelling tasks. Right columns display mean accuracy (n = 80) and whether the item was included in 100-item match and label or 65-item match and 48-item label.
(XLSX)
