# Supplementary material for: New Tests to Measure Individual Differences in Matching and Labelling Facial Expressions of Emotion, and Their Association with Ability to Recognise Vocal Emotions and Facial Identity
Source: PLoS One. 2013 Jun 28;8(6):e68126. doi: 10.1371/journal.pone.0068126 (PMC3695959; doi:10.1371/journal.pone.0068126)
Supplement: Results S1 — Analysis of other versions of the emotion-matching and emotion-labelling tasks (144, 65, and 48 item versions). (PDF) [file pone.0068126.s001.pdf]

<u>Results S1.</u> Analysis of other versions of the emotion-matching and emotion-labelling tasks (144-, 65-, and 48-item versions).

1. Original 144-item emotion-matching and emotion-labelling tasks

Shapiro-Wilks tests confirmed that scores on the emotion-matching task (*W* = .98, *df* = 80, *p* = .18) and the emotion-labelling task (*W* = .98, *df* = 80, *p* = .30) were normally distributed (Figures A and B). The emotion-matching data were negatively skewed (skew = .57, SE = .27, z = 2.10, p = .04) but there were no univariate outliers. There was no evidence of skew in the emotion-labelling task (skew = .42, SE = .27, z = 1.55, p = .12) but a potential univariate outlier, with a z-score of -3.36. The performance of this participant appears to reflect generally poor face emotion recognition ability (emotion-matching task z = -1.74) rather than general ability (CFIT z = -0.78; CFMT z = .02) and as such the data from the participant was retained. Mahalanobis distances revealed no multivariate outliers, based on a criterion of p < .001. Performance on both tests, with average accuracy of 74%, was neither at floor or ceiling (Matching: 106.70/144, SD = 9.13, chance = 48; Labelling: 106.59/144, SD = 7.31, chance = 24). There was no significant difference between female (*M* = 108.06, *SD* = 9.93) and male (*M* = 104.31, *SD* = 7.06) participants on the emotion-matching task (*t*(78) = 1.79, *p* = .08), however females (*M* = 108.00, *SD* = 7.47) performed significantly better than males (*M* = 104.10, *SD* = 6.42) on the emotion-labelling task, *t*(78) = 2.36, *p* = .02.

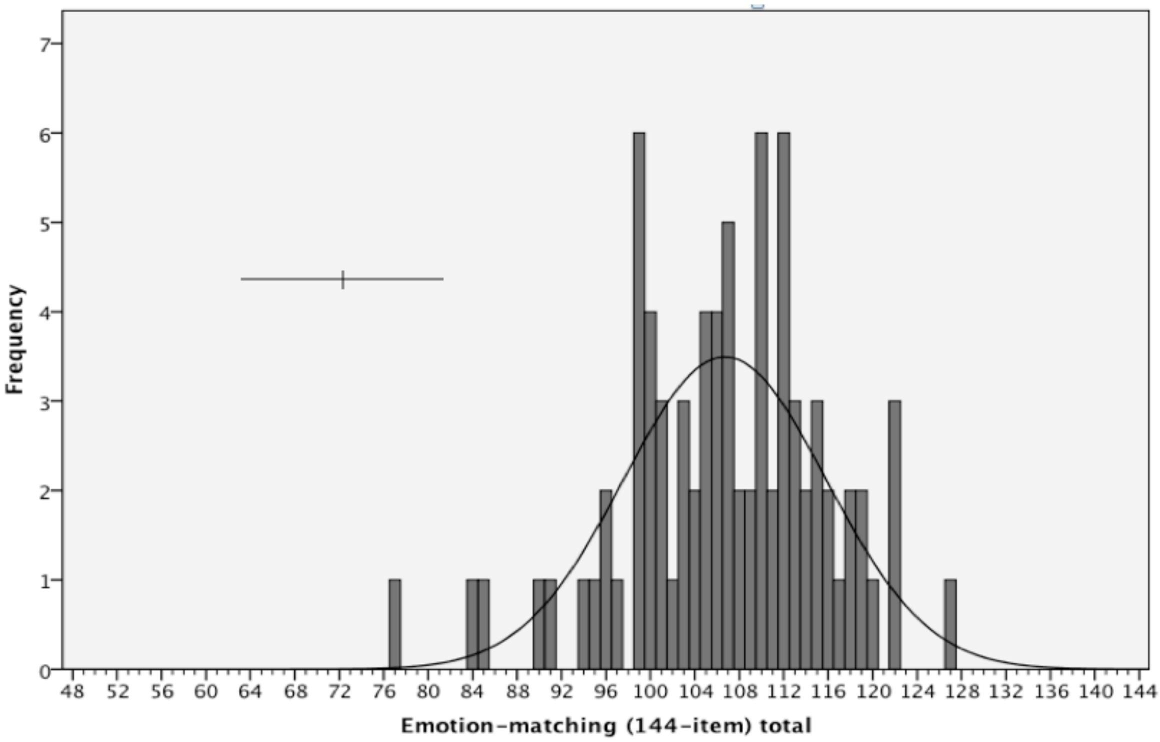

Figure A. Frequency distribution for scores the 144-item Emotion-matching task (chance performance = 48, N = 80). Error bar depicts +/- 95% confidence interval.

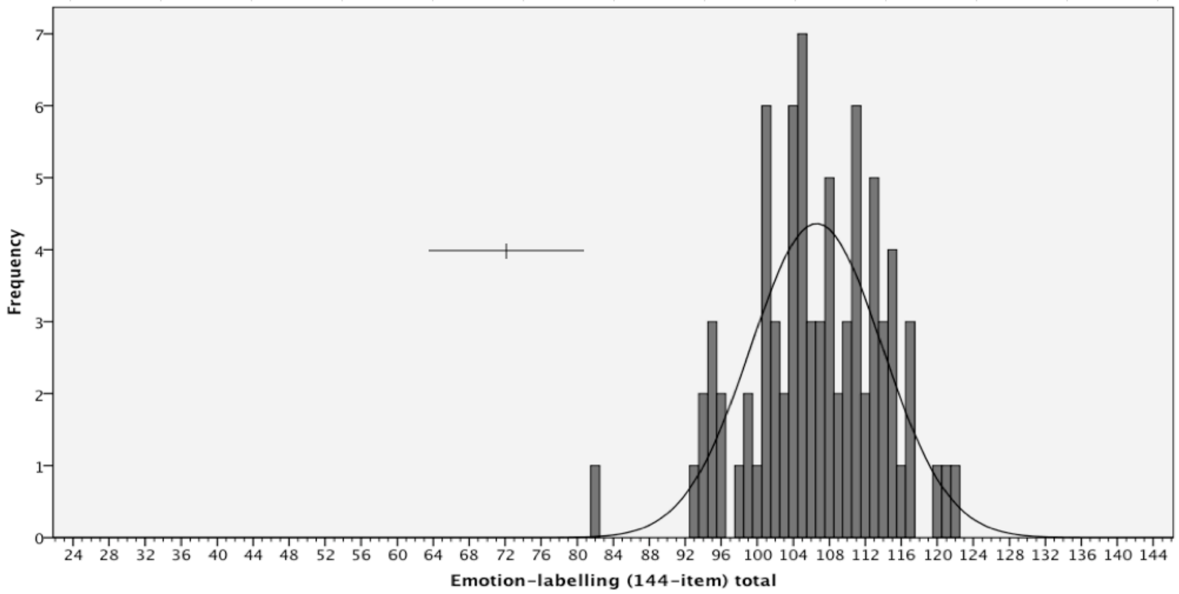

Figure B. Frequency distribution for scores the 144-item Emotion-labelling task (chance performance = 24, N = 80). Error bar depicts +/- 95% confidence interval.

2. 65-item emotion-matching and 48-item emotion-labelling tasks

For each test, we also progressively removed target faces until no further improvement in reliability emerged and the highest level of reliability was obtained (Table A). At this stage there were 65 items in the emotion-matching task and 48 items in the emotion-labelling task[1]. Performance was neither at floor or ceiling (emotion-matching: 50.28/65, SD = 6.99, chance = 22; emotion-labelling: 37.40/48, SD = 5.60, chance = 8; Table A), however the scores were not normally distributed (emotion-matching W = .96, df = 80, p = .01; emotion-labelling W = .96, df = 80, p= .02) and there was evidence of negative skew for the emotion-matching task (skew = -.74, SE = .27, z = 2.76, p = .009) (see Figures C and D). As such, the 100-item tests are preferred for assessing individual differences in the ordinary population, whereas these short and most reliable tests might be useful for use with patients, especially when under time pressure.

[1] Cronbach's alpha is also highest for the 48-item emotion-labelling test when data from the outlier is excluded.

Table A. Range, Mean (SD) and Cronbach's alpha for 144-item Emotion-matching and –labelling tasks and 65 -item Emotion-matching and 48-item Emotion-labelling tasks.

Task	Observed Range %		Total (N=80)		Female (n=51)		Male (n=29)	
	Min.	Max.	M (SD)	α	M (SD)	α	M (SD)	α
144 -item Emotion-matching	53.47	88.19	74.10 (6.34)	0.74	75.04 (6.89)	0.78	72.44 (4.90)	0.55
144-item Emotion-labelling	56.94	84.72	74.02 (5.08)	0.64	75.00 (5.19)	0.66	72.29 (4.46)	0.54
65 -item Emotion-matching	46.15	98.46	77.35 (10.75)	0.82	78.85 (10.81)	0.83	74.69 (10.29)	0.80
48-item Emotion-labelling	47.92	95.83	77.92 (11.67)	0.80	80.43 (10.78)	0.79	73.49 (12.04)	0.79

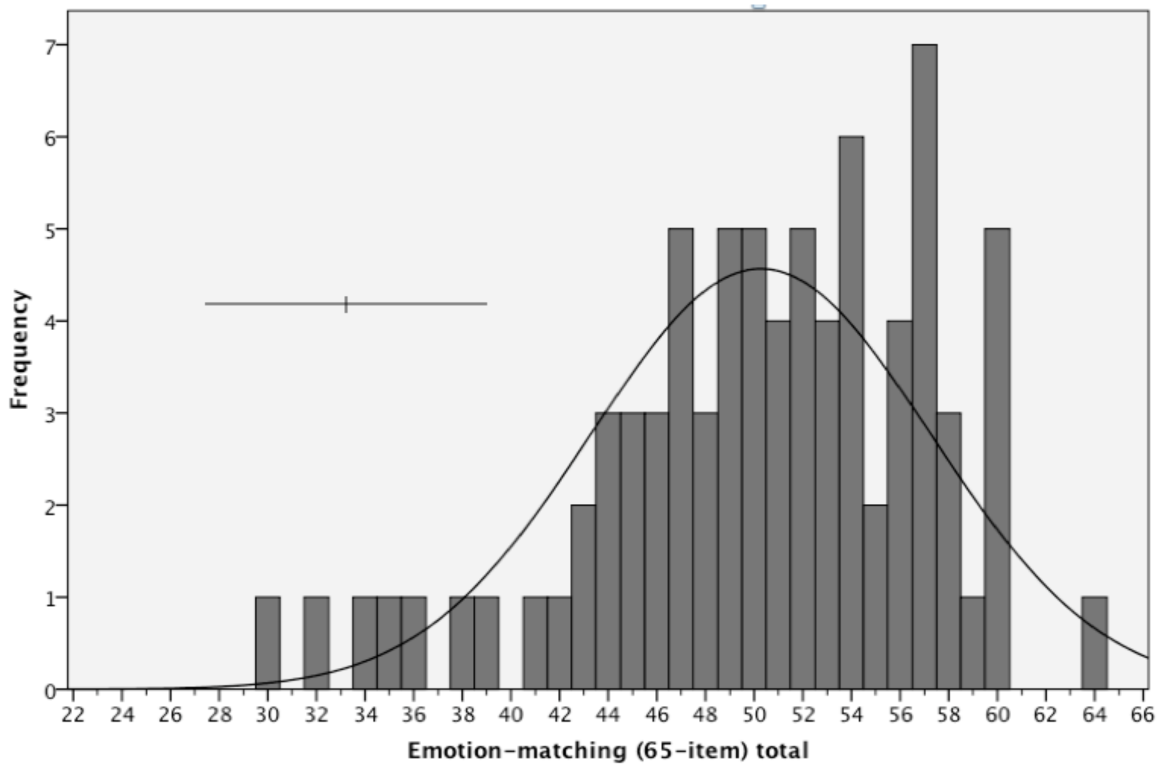

Figure C. Frequency distribution for scores the 65-item Emotion-matching task (chance performance = 22, N = 80). Error bar depicts +/- 95% confidence interval.

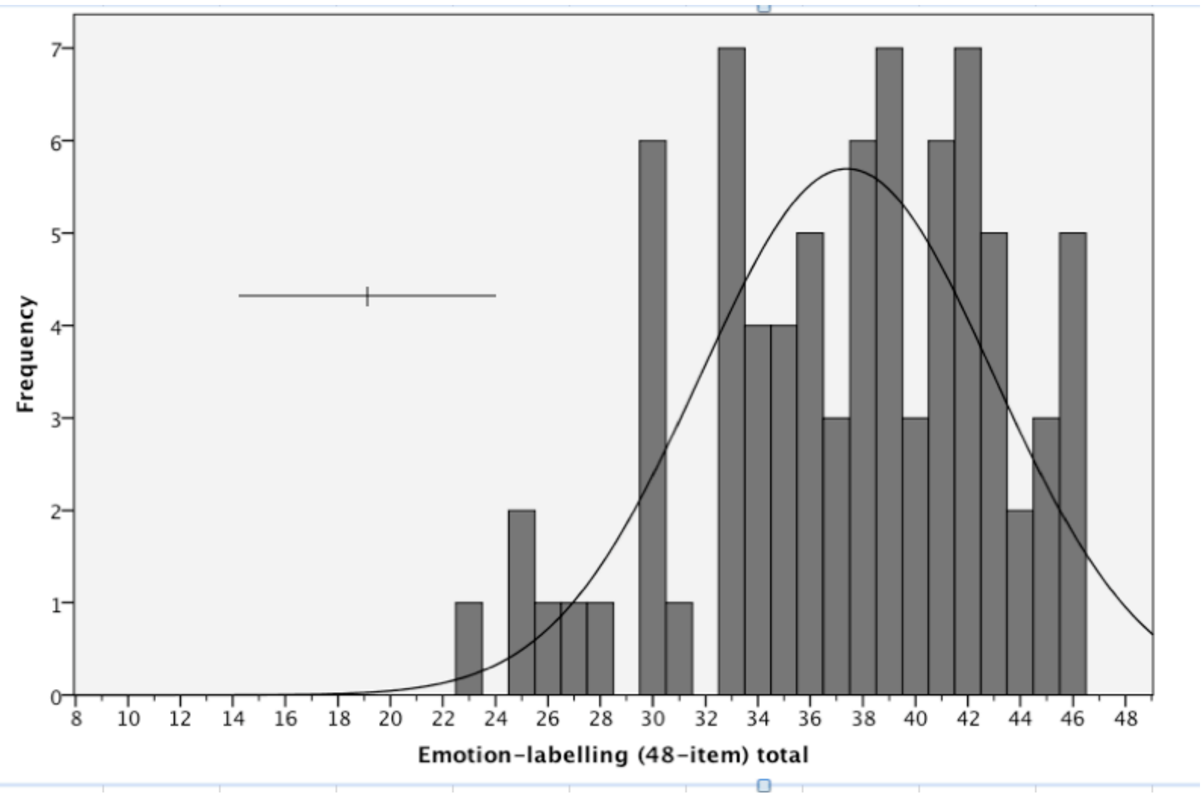

Figure D. Frequency distribution for scores the 48-item Emotion-labelling task (chance performance = 8, N = 80).

Table B. Pearson's r and Spearman's rho correlations between the emotion-matching and –labelling tasks and the vocal emotion labelling task and the Cambridge Face Memory Test

**Task**	Vocal emotion labelling	Cambridge Face Memory Test
144 -item Emotion-matching	r = .19, rho = .17 (max = .71)	r = .36**, rho = .30**(max = .82)
144-item Emotion-labelling	r = .25*, rho = .19 (max = .66)	r = .25*, rho = .25* (max = .76)
65 -item Emotion-matching	r = .21, rho = .19 (max = .75)	r = .37**, rho = .36** (max = .86)
48-item Emotion-labelling	r = .30**, rho = .25* (max = .74)	r = .22, rho = .20 (max = .85)

** p < .01; * p < .05
