# Supplementary material for: New Tests to Measure Individual Differences in Matching and Labelling Facial Expressions of Emotion, and Their Association with Ability to Recognise Vocal Emotions and Facial Identity
Source: PLoS One. 2013 Jun 28;8(6):e68126. doi: 10.1371/journal.pone.0068126 (PMC3695959; doi:10.1371/journal.pone.0068126)
Supplement: Results S2 — Reliability for separate expressions in the 144-item and 100-item emotion-matching and –labelling tasks. (PDF) [file pone.0068126.s002.pdf]

Results S2. **Performance on 100-item matching task by different sample.**

Participants
Participants were 167 Caucasian undergraduate students (119 female) aged between 18 and 43 (M = 20.37, SD = 3.98). Four participants did not appear to follow task instructions, either responding with non-target keys or responding very quickly, and scored at or below chance, and were removed from further analyses. All participants completed the experiment as part of a cognitive psychology course requirement.

Stimuli and Procedure
Participants completed the final 100-item version of the emotion-matching task. Procedure and stimuli were identical to those previously described, with the exception that the face stimuli were reduced to 75% of the original size (approximately 6.75° x 4.5°).

Results
Rating accuracy ranged from 47% to 87%, with a mean of 71.55% (SD = 7.81%). There was no significant difference in performance between male (M = 70.94, SD = 7.73) and female participants (M = 71.80, SD = 7.86), $t(161)$ = .64, p = .52. A Shapiro-Wilks test revealed that the data were not normally distributed (W = .96, df = 163, p < .001) and there was significant negative skew (skew = -.73, SE = .19, z = 3.82, p < .001). Cronbach's alpha indicated moderate internal consistency (α = .74; Males: α = .67; Females: α = .65).

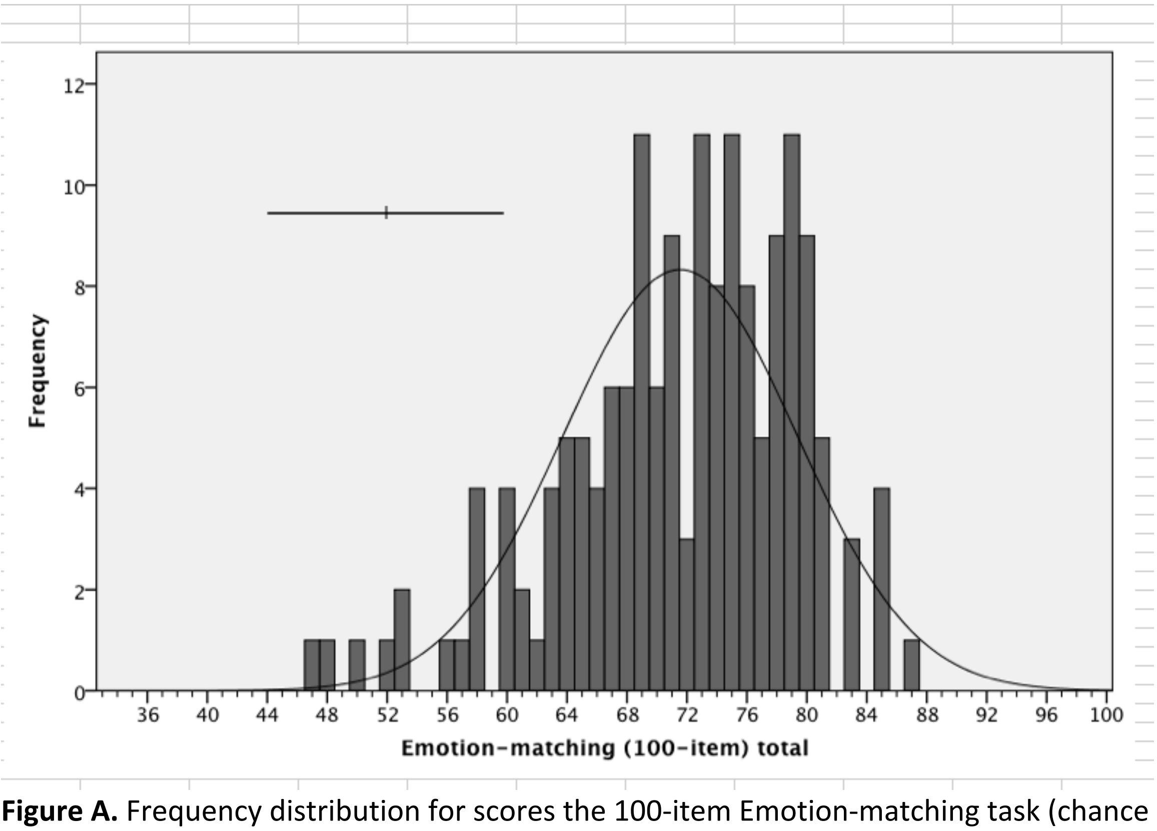

Figure A. Frequency distribution for scores the 100-item Emotion-matching task (chance performance = 33, N = 163). Error bar depicts +/- 95% confidence interval.
