# Supplementary material for: New Tests to Measure Individual Differences in Matching and Labelling Facial Expressions of Emotion, and Their Association with Ability to Recognise Vocal Emotions and Facial Identity
Source: PLoS One. 2013 Jun 28;8(6):e68126. doi: 10.1371/journal.pone.0068126 (PMC3695959; doi:10.1371/journal.pone.0068126)
Supplement: Results S3 — Performance on 100-item matching task by different sample. (PDF) [file pone.0068126.s003.pdf]

<u>Results S3.</u> Reliability for separate expressions in the 144-item and 100-item emotion-matching and –labelling tasks.

Table A. Cronbach's alpha (α) for each of the separate expressions in the 144-item and 100-item emotion-matching tests.

	**144-item test (N = 80)**				**100-item test (N = 243)[a]**			
**Expression**	**Range (%)**	**M (SD)**	**α**	**# items**	**Range (%)**	**M (SD)**	**α**	**# items**
Anger	58.33–100	79.74 (10.16)	0.50	24	35.00–100	77.65 (11.34)	0.48	20
Disgust	33.33–91.67	65.05 (12.66)	0.52	24	21.43–100	61.96 (15.66)	0.41	14
Fear	33.33–87.50	67.03 (10.15)	0.29	24	25.00–100	67.22 (14.03)	0.21	12
Happiness	62.50–100	87.24 (8.13)	0.41	24	40.91–100	84.14 (11.03)	0.59	22
Sadness	45.83–87.50	67.40 (10.01)	0.27	24	29.41–100	65.04 (13.46)	0.43	17
Surprise	58.33–95.83	78.13 (8.42)	0.20	24	33.33–100	77.96 (11.64)	0.36	15

[a] Sample consists of N = 80 reported in the main text and N = 163 who completed the 100-item matching test reported in Supplementary Materials C.

Table B. Cronbach's alpha (α) for each of the separate expressions in the 144-item and 100-item emotion-labelling tests.

	**144-item test (N = 80)**				**100-item test (N = 80)**			
**Expression**	**Range (%)**	**M (SD)**	**α**	**# items**	**Range (%)**	**M (SD)**	**α**	**# items**
Anger	41.67–95.83	79.11 (11.65)	0.65	24	35.00–100	83.56 (12.38)	0.68	20
Disgust	16.67–87.50	57.55 (14.45)	0.66	24	14.29–100	72.77 (18.28)	0.66	14
Fear	4.17–91.67	49.06 (16.48)	0.72	24	8.33–100	64.38 (20.92)	0.67	12
Happiness	75.00–100	95.99 (5.29)	0.49	24	72.73–100	96.59 (5.31)	0.52	22
Sadness	54.17–100	80.31 (10.92)	0.62	24	47.06–100	83.60 (13.11)	0.65	17
Surprise	50.00–100	82.08 (11.48)	0.62	24	53.33–100	86.75 (12.18)	0.54	15

Note: Not all the items in the 144-item tests are considered valid depictions of the intended emotion.
